# *FOXP3* Promoter Demethylation Reveals the Committed Treg Population in Humans

**DOI:** 10.1371/journal.pone.0001612

**Published:** 2008-02-20

**Authors:** Peter C. J. Janson, Malin E. Winerdal, Per Marits, Magnus Thörn, Rolf Ohlsson, Ola Winqvist

**Affiliations:** 1 Department of Medicine, Clinical Allergy Research Unit, Karolinska Institutet, Stockholm, Sweden; 2 Department of Surgery, South Stockholm General Hospital, Stockholm, Sweden; 3 Department of Development and Genetics, Evolution Biology Center, Uppsala University, Uppsala, Sweden; Université de Toulouse, France

## Abstract

**Background:**

Naturally occurring thymus derived regulatory T cells (Tregs) are central in the maintenance of self-tolerance. The transcription factor FOXP3 is crucial for the suppressive activity of Tregs and is considered the most specific marker for this population. However, human non regulatory T cells upregulate FOXP3 transiently upon activation which calls for other means to identify the Treg population. Since epigenetic mechanisms are involved in the establishment of stable gene expression patterns during cell differentiation, we hypothesized that the methylation profile of the *FOXP3* promoter would allow the distinction of truly committed Tregs.

**Methodology/Principal Findings:**

Human CD4^+^CD25^hi^ Tregs displayed a demethylated *FOXP3* promoter (1.4%±0.95% SEM methylated) in contrast to CD4^+^CD25^lo^ T cells which were partially methylated (27.9%±7.1%). Furthermore, stimulated CD4^+^CD25^lo^ T cells transiently expressed FOXP3 but remained partially methylated, suggesting promoter methylation as a mechanism for regulation of stable FOXP3 expression and Treg commitment. In addition, transient FOXP3 expressing cells exhibited suppressive abilities that correlate to the methylation status of the *FOXP3* promoter. As an alternative to bisulphite sequencing, we present a restriction enzyme based screening method for the identification of committed Tregs and apply this method to evaluate the effect of various culturing conditions. We show that a partial demethylation occurs in long-term cultures after activation, whereas the addition of TGF-β and/or IL-10 does not induce any additional change in methylation level.

**Conclusions/Significance:**

The unique *FOXP3* promoter methylation profile in Tregs suggests that a demethylated pattern is a prerequisite for stable FOXP3 expression and suppressive phenotype. Presently, FOXP3 is used to identify Tregs in several human diseases and there are future implications for adoptive Treg transfer in immunotherapy. In these settings there is a need to distinguish true Tregs from transiently FOXP3^+^ activated T cells. The screening method we present allows this distinction and enables the identification of cells suitable for *in vitro* expansions and clinical use.

## Introduction

Human immunity is an intriguingly complex balance of self defence versus autoreactivity. Naturally occurring thymus-derived Tregs are a subpopulation of T cells which play a central role as regulators of immune response. The transcription factor FOXP3 has been linked to the suppressive phenotype of both human (FOXP3) and murine (Foxp3) Treg populations [1]–[4]. Mutations in the human *FOXP3* gene causes the disease Immune dysregulation, Polyendocrinopathy, Enteropathy, X-linked syndrome (IPEX) [5], and the *Foxp3* mutant *Scurfy* mouse model displays a similar pathology involving dysregulated CD4^+^ T cell infiltration and activation [6]–[8]. Although transient expression of Foxp3 has been observed in murine activated T cells [9], Foxp3 is not only considered a specific marker for the Treg population but it is also required and sufficient for Treg development in the murine setting [1], [3]. In humans however, recent reports indicate that FOXP3 may not be as specific as its murine counterpart. Just as CD25, FOXP3 is transiently upregulated in human CD4^+^CD25^lo^ T cells upon activation [10]–[15] and although this FOXP3 expression is associated with hyporesponsiveness and decreased cytokine production, results regarding the suppressive ability of these cells differ [14], [16], [17]. The transduction of CD45RA^+^CD4^+^CD25^lo^ with a FOXP3-encoding retrovirus resulted in significant FOXP3 expression, however this was not sufficient to induce a suppressive phenotype or upregulation of Treg surface markers [16]. Presently, FOXP3 is used to identify Treg cells in several human diseases including autoimmune conditions [18], [19], infections [20], [21] and cancer [22]. In these settings, there is a need for Treg markers able to distinguish this cell population from activated T cells.

Considering the importance of FOXP3 in the control of immune responses, the factors which in turn control FOXP3 become of interest. The *FOXP3* promoter region was recently described and shown to be accessible for the transcription machinery in both CD4^+^CD25^hi^ and CD4^+^CD25^lo^ T cells [11]. It was also found to contain binding sites for nuclear factor of activated T cells (NFAT) and activator protein 1 (AP-1), transcription factors which are well-established mediators of T cell activation, in agreement with the possibility of FOXP3 transcription in activated CD4^+^CD25^lo^ T cells [11] as well as the fact that Tregs need to be activated in order to acquire suppressor function [23].

Epigenetic control is a well-established means of gene regulation within the immune system. Mechanisms such as histone modifications and DNA methylation carefully govern cell fate decisions in developing lymphocytes [24]–[26]. The epigenetic mechanisms involved in Th1 and Th2 development are well described, whereas the mechanisms controlling Treg development are just beginning to be explored. Mice studies have focused on a conserved element in intron one of the *Foxp3* gene which shows transcriptional activity and differential methylation between CD4^+^CD25^−^ and T regulatory cells [27], [28]. However, the methylation status of the human *FOXP3* promoter has not yet been examined. The observation that human NK cells show increased FOXP3 expression when treated with the demethylating reagent 5-aza-2′-deoxycytidine strongly supports the assumption that FOXP3 expression is methylation dependent [29].

In this report, we investigated the epigenetic status of the human *FOXP3* promoter region, and correlated CpG methylation to FOXP3 expression with the aim to identify putative methylation sites to be used as Treg markers. We report Treg associated demethylation in the *FOXP3* promoter and identify a suitable site for methylation based screening of Treg commitment. Furthermore, we evaluate the demethylating abilities of several different stimuli and culturing conditions. In conclusion, complete demethylation of the human FOXP3 promoter is confined to committed Tregs and thus analysis of promoter methylation enables the identification of cells suitable for *in vitro* expansions and clinical use.

## Results

### Isolation and Analysis of Cell populations

**Figure 1:**
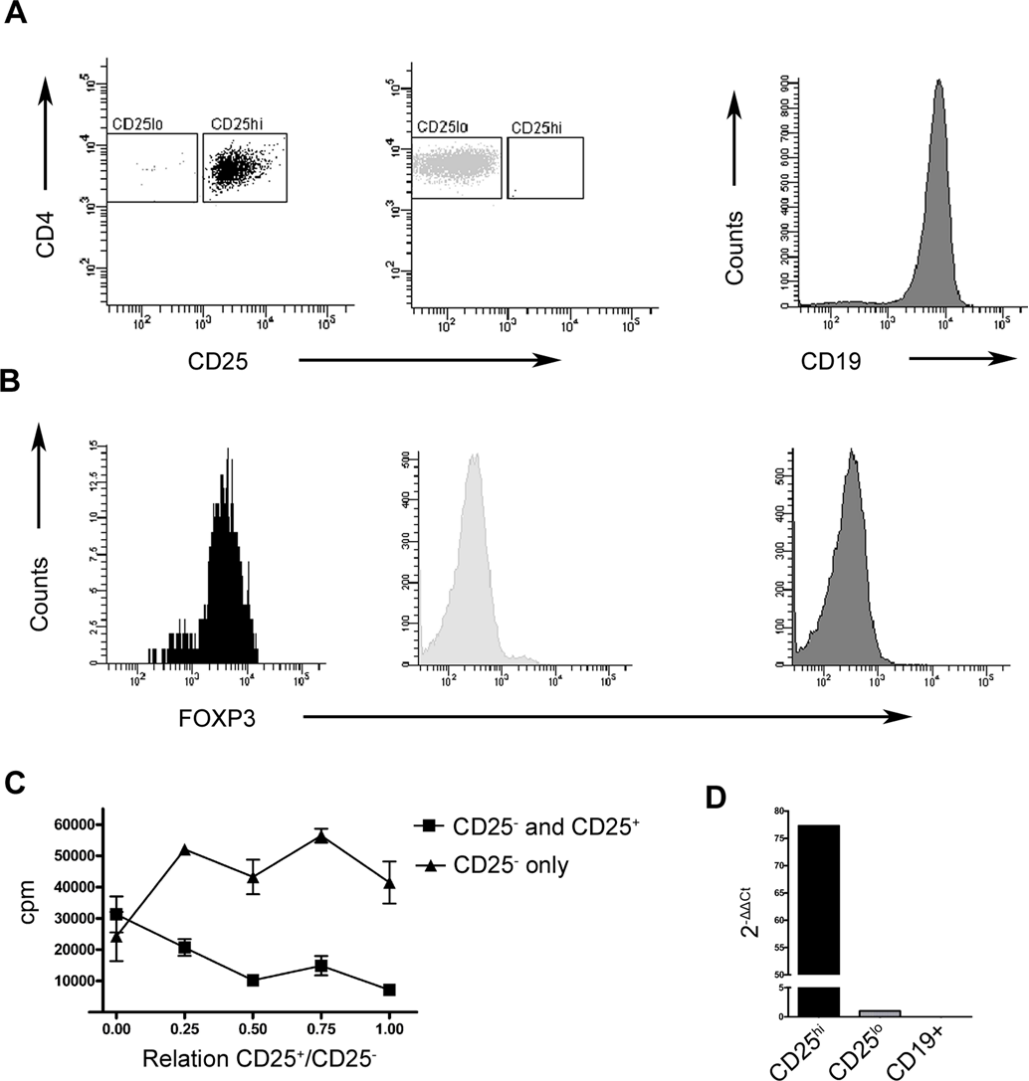
FOXP3 expression in sorted cell populations and confirmation of Treg function. (A) The sorted CD4^+^CD25^lo^, CD4^+^CD25^hi^ and CD19^+^ cells were analyzed by flow cytometry. (B) FOXP3 expression in the sorted populations as determined by intracellular flow cytometry. Plots showing results from one representative donor out of four analyzed, except for plot showing FOXP3 expression in CD19^+^ cells where one single donor was analyzed (C) Sorted CD4^+^CD25^+^ cells suppress the proliferation of CD4^+^CD25^−^ cells. CD4^+^CD25^−^ cells were activated with CD3 and CD28 antibodies together with CD4^−^ feeder cells and increasing numbers of CD4^+^CD25^+^ cells in triplicate samples. Proliferation was measured as incorporation of [^3^H]Thymidine for 18 hours, here illustrated as counts per minute (cpm) on the y-axis. The CD4^+^CD25^+^ to CD25^−^ cell ratio is displayed on the x-axis. Squares indicate coculture of CD25^−^ and CD25^+^ cells. Triangles indicate control samples with only CD4^+^CD25^−^ cells. (D) *FOXP3* mRNA expression of sorted cell populations. *FOXP3* mRNA was measured by real-time PCR in FACS sorted CD4^+^CD25^hi^, CD4^+^CD25^lo^ and CD19 cells. Data was normalized to the expression in CD4^+^CD25^lo^ cells using the 2^−ΔΔCt^ method and RPII as housekeeping gene. Data represent mean of triplicate samples from one single donor.

PBMC from healthy blood donors were recovered from buffy coats. Subsequently, CD4^+^ T cells were isolated and analyzed with respect to expression of CD4, CD25 and FOXP3 (Figure 1A–B). In agreement with previous results [30], FOXP3 expression was prominent in the CD4^+^CD25^hi^ T cell population, with very few CD4^+^CD25^lo/int^ cells being FOXP3^+^. As a control the CD19^+^ B cell population was found to be FOXP3^−^ (Figure 1A–B). After cell sorting, cell populations were >95% pure as demonstrated with FACS (Figure 1A).

### Confirmation of Treg Function and Analysis of FOXP3 Expression

Next the functionality of sorted Tregs was tested. The ability of sorted CD4^+^CD25^+^ cells to suppress the proliferation of anti-CD3 and anti-CD28 activated CD4^+^CD25^−^ cells was investigated by co-culture experiments measuring proliferation by [^3^H]Thymidine incorporation (Figure 1C). Suppressive effect of the CD4^+^CD25^+^ Treg cells was noted already at the ratio of one to four CD4^+^CD25^+^ Treg cells to CD4^+^CD25^−^ T responder cells in consistency with previous reports [23].

To confirm the Treg nature of sorted CD4^+^CD25^+^ cells *FOXP3* mRNA expression was measured by quantitative real-time PCR. The levels of *FOXP3* transcripts were 75-fold elevated in isolated CD4^+^CD25^hi^ cells when compared to expression in CD4^+^CD25^lo^ cells. Conversely, the CD19^+^ B cells displayed no detectable *FOXP3* mRNA expression (Figure 1D). Thus sorted CD4^+^CD25^hi^ T cells demonstrate a functional suppressive phenotype and display a high *FOXP3* mRNA expression.

### Identification of evolutionarily conserved CpG's in the FOXP3 promoter

**Figure 2:**
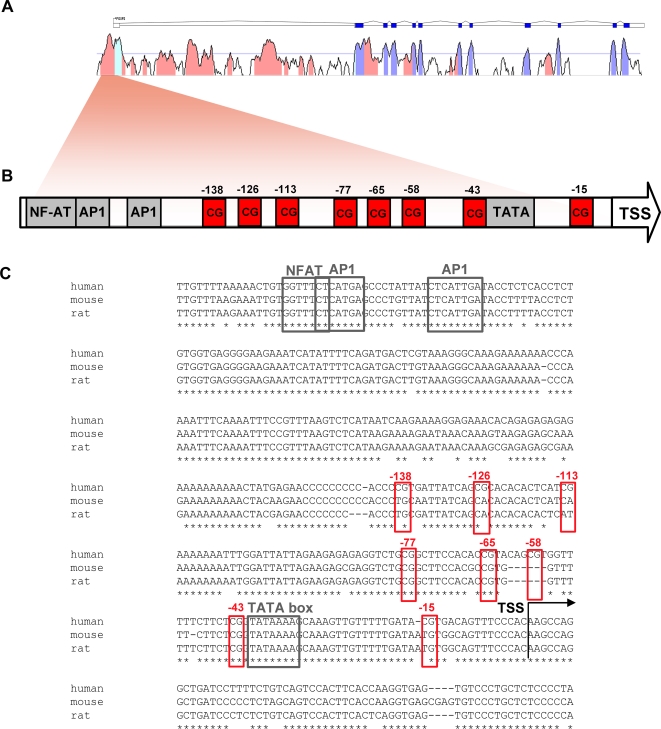
Identification of cross-species conserved *FOXP3* promoter elements. (A) *FOXP3* transcript and m-Vista alignment showing conservation between human and mouse genomic sequences. Dark blue boxes display exons, outlined boxes are UTR's. Conserved regions are in red and conserved regions corresponding to exons are in light blue. (B) Schematic view of the conserved region upstream of the transcription start site indicating the location of promoter elements and CpG dinucleotides. (C) Clustal W alignment of human, mouse and rat genomic sequences showing a detailed view of the conservation at the *FOXP3* core promoter. The broken arrow shows the position of the human transcription start site. The location of the TATA box is indicated by a black box. Red boxes indicate the CpG dinucleotides analyzed in this study. Transcription factor binding sites are marked with grey boxes.

The functional human *FOXP3* promoter has been previously defined [11]. The region preceding the 5′ untranslated region (UTR) is highly conserved and contains important promoter elements such as TATA, GC and CAAT boxes as well as binding sites for NFAT and AP-1, well-established mediators of T cell activation (Figure 2B). The high degree of conservation of such cis-acting motifs in this region (Figure 2A) indicated that it might be under epigenetic influence. Therefore we investigated the *FOXP3* promoter region with regards to CpG dinucleotides using ClustalW alignment comparing human, mouse and rat sequences (Figure 2C). The whole promoter region showed a mouse to man conservation of 87% (online supplemental material Figure S1), whereas the CpG containing region was 92% conserved, stressing its evolutionary importance (online supplemental material Figure S2). Moreover, ClustalW alignment determined that out of eight tightly positioned CpG dinucleotides just upstream of the transcription start site, positions −43, −65 and −77 were totally conserved between species. As these CpGs mapped just downstream of the binding sites for the transcription factors NFAT and AP-1 (Figure 2B–C), a special interest in these positions was rendered.

### Methylation Status of the FOXP3 promoter

**Figure 3:**
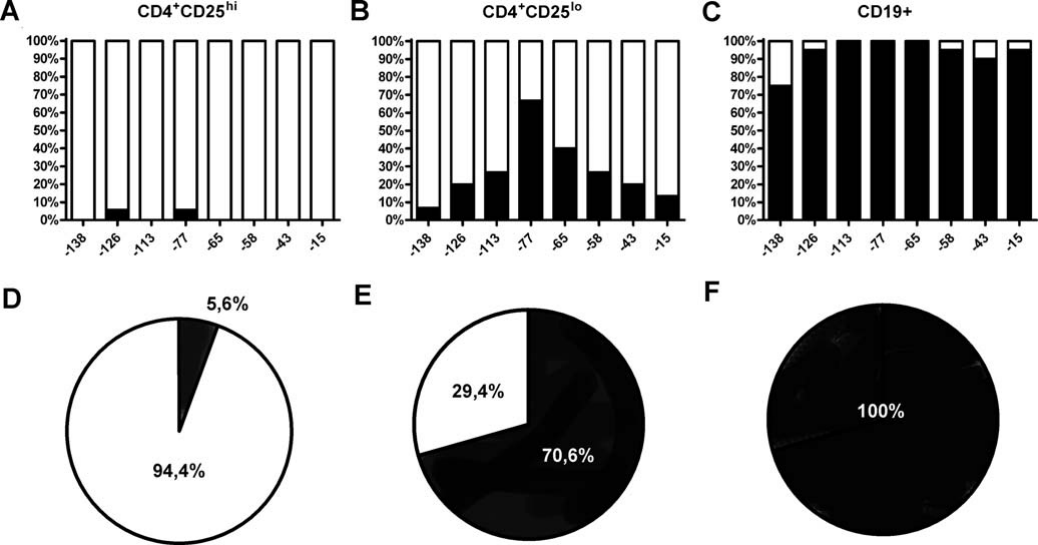
Tregs are unmethylated at the *FOXP3* promoter region. Bars in (A) to (C) indicate the percentage of methylated (black) and unmethylated (white) cells at each CpG position (specified on x-axis labels as relative the transcription start site) in (A) CD4^+^CD25^hi^, (B) CD4^+^CD25^lo^ and (C) CD19^+^ cell populations isolated by FACS from one male donor. (D) to (F) Methylation grade of the most discriminating CpG position (−77). Percentage methylated (filled) versus unmethylated (open) CpGs in the (D) CD4^+^CD25^hi^, (E) CD4^+^CD25^lo^ and (F) CD19^+^ cells.

The putative methylation sites within the promoter region prompted the investigation of the methylation status of these positions by bisulphite sequencing. Cells were isolated from male donors only in order to avoid possible artefacts due to random X chromosome inactivation in females. CD4^+^CD25^hi^ FOXP3^+^ T cells (n = 18) were found to contain overall unmethylated CpGs (Figure 3A), whereas CD19^+^FOXP3^−^ B cells (n = 20) displayed a fully methylated configuration (Figure 3C). The CD4^+^CD25^lo^ population (n = 17) displayed an intermediate level of methylation (Figure 3B), and differed significantly from the CD4^+^CD25^hi^ FOXP3^+^ population at CpG positions −113, −77, −65, and −58 as detailed in Table 1. Interestingly, the difference was most pronounced (p<0.001) at the evolutionary conserved site CpG position −77 (Figure 3D–F). The p-values of each position clearly demonstrate the differences between the populations (Table 1).

**Table 1:** P-value for each CpG position in sorted cells.

	*−138*	*−126*	*−113*	*−77*	*−65*	*−58*	*−43*	*−15*
*CD25^hi^ vs CD25^lo^*	0.47	0.15	<0.05	<0.001	<0.01	<0.05	0.10	0.23
*CD25^lo^ vs CD19^+^*	<0.001	<0.001	<0.001	<0.05	<0.001	<0.001	<0.001	<0.001
*CD25^hi^ vs CD19^+^*	<0.001	<0.001	<0.001	<0.001	<0.001	<0.001	<0.001	<0.001

Overall the CD4^+^CD25^hi^ Treg cells demonstrated a low level of methylation (1.4%±0.95% SEM) at the CpG sites investigated. In contrast, the CD4^+^CD25^lo^ population was methylated to 27.9%±7.1% and the CD19^+^ B cells were almost completely methylated (93.8%±1.7%). Taken together these data show significant differences in the degree of methylation in the *FOXP3* promoter region, where the Treg population is unmethylated and CD4^+^CD25^lo^ T cells and CD19^+^ B cells demonstrate increasing levels of methylation.

### COBRA Based Methylation Screening

**Figure 4:**
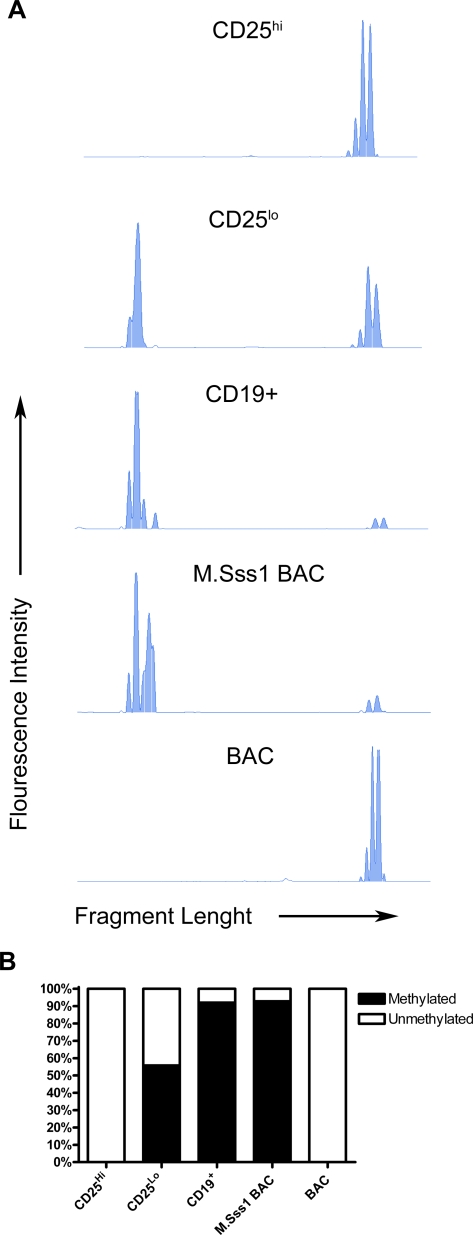
Restriction enzyme based analysis of the methylation status at the *FOXP3* −77 reporter position. PCR product from bisulphite treated DNA was digested with the restriction enzyme Aci1, which only cleaves DNA at position −77 in the *FOXP3* promoter when DNA originally was methylated. Digestion was followed by GeneScan analysis. (A) Electropherograms showing peaks from the Aci1 digestion product. Amounts of digested and undigested product were calculated as area under the curve. Shown are results from one representative donor out of three analyzed, except for electropherogram obtained from CD19^+^ cells where one single donor was analyzed. Digestion product from methylated and unmethylated BAC was analyzed three times with similar results. (B) Filled bars indicate percentage methylated, and open bars percentage unmethylated −77 sites. Methylase treated and unmodified BACs were used as controls.

Bisulphite sequencing is in itself both time-consuming and limited by the number of clones analyzed in its ability to reflect the true methylation status of a cell population. Therefore, we developed a COBRA based method of screening the methylation status at the evolutionary conserved CpG position −77 in the *FOXP3* promoter region. The method was tested on *ex vivo* collected cell populations, with methylase treated and untreated BAC as controls (Figure 4). Multiple peaks (separated by less than 3 bp) observed in the electropherograms are likely due to exonuclease activity and/or star activity during the digestion. The bisulphite treated PCR product obtained from CD4^+^CD25^hi^ Tregs remained undigested (Figure 4A top panel) in agreement with the results derived from the unmethylated BAC control (Figure 4A bottom panel). The methylated BAC was >90% digested as well as the methylated promoter region derived from sorted CD19^+^ B cells (Figure 4A third panel from the top). In concordance with the sequencing data CD4^+^CD25^lo^ T cells displayed a partially unmethylated −77 CpG position (Figure 4A second panel from the top). Thus investigating the −77 reporter CpG position (Figure 4B) mirrors the results obtained by bisulphite sequencing confirming the validity of the method.

### FOXP3 Expression in Activated T cell Populations

**Figure 5:**
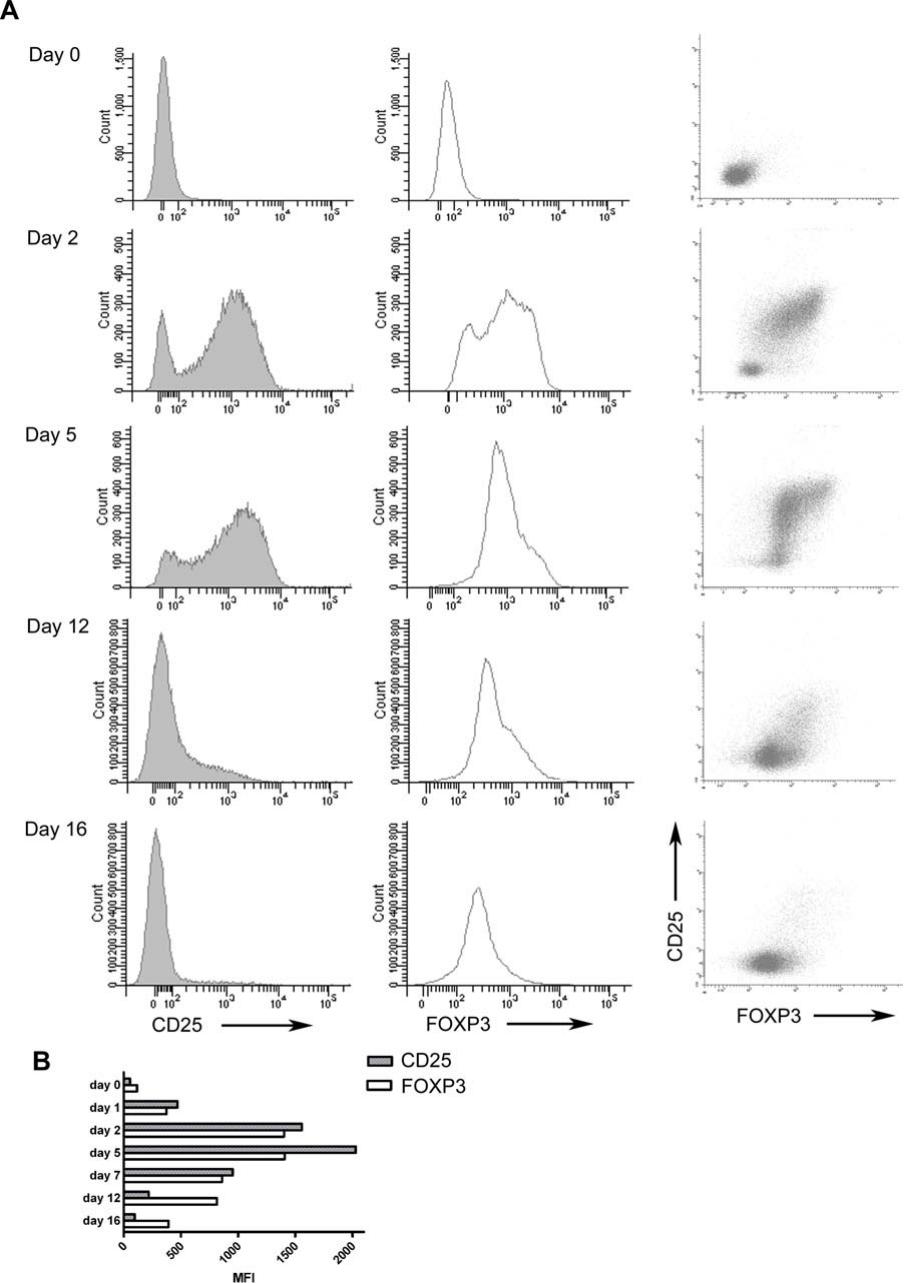
Transient expression of FOXP3 and CD25 in stimulated CD4^+^CD25^lo^ cells from male donors. Plots showing results from one representative donor out of four analyzed. (A) FACS analysis of CD4^+^CD25^lo^ cells stimulated at day 0 with CD3/CD28 Dynabeads (proportion cell∶beads 1∶1) in presence of 180 U IL-2. Stimuli removed after 48 h and cells followed for an additional 2 weeks with regards to their expression of CD25 (left) and FOXP3 (middle). Left column demonstrating the relationship between CD25 (y-axis) and FOXP3 (x-axis) expression. (B) Expression of CD25 (grey filled bars) and FOXP3 (open bars) in stimulated cells as described above, displayed as mean fluorescent intensity (MFI).

Previously, activated CD4^+^CD25^lo^ cells have been shown to transiently upregulate FOXP3 [13], [14]. Therefore, we stimulated CD4^+^CD25^lo^ T cells with anti-CD3/CD28 Dynabeads for 48 hours and monitored the expression of FOXP3 and CD25 up to day 16 of culture (Figure 5A). Mean fluorescent intensity (MFI) for FOXP3 and CD25 expression at each time point was calculated (Figure 5B). CD25 showed a faster response to TCR stimulation in comparison to FOXP3. Maximum MFI of FOXP3 was recorded day 5 of culture after which it decreased. However, the cells still displayed some residual FOXP3 expression at day 16. These results confirm that FOXP3 expression occurs in CD4^+^CD25^lo^ T cells upon stimulation, and thus is not solely limited to the Treg population in humans.

### Methylation of the *FOXP3* promoter during T cell activation

**Figure 6:**
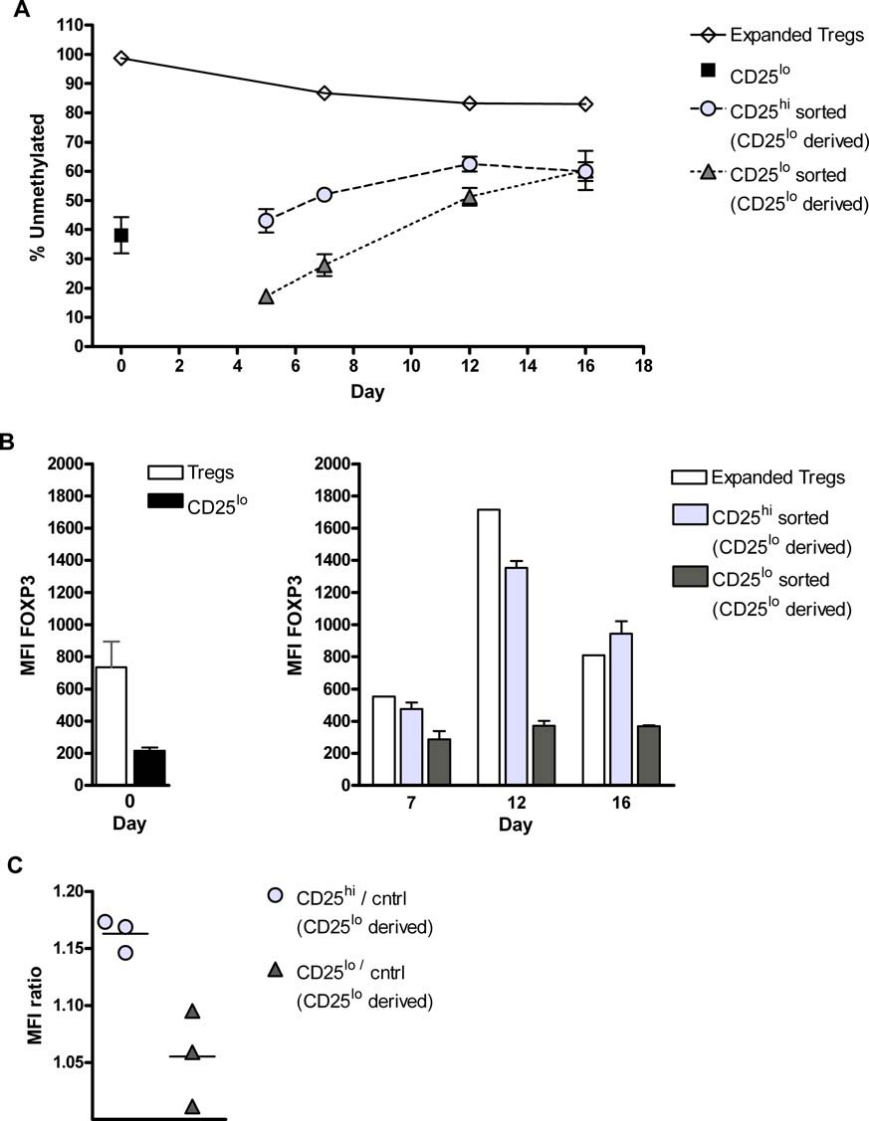
Activated CD25^lo^ T cells demethylate during proliferation and exert suppressive activity *in vitro*. The methylation level of activated cells derived from the CD25^lo^ population was monitored with the COBRA based analysis described in Figure 4. (A) Cultures from three separate donors were setup and stimulated as in Figure 5, after which the methylation status in the CD25^hi^ and CD25^lo^ fraction was monitored for 16 days. Cultures of T regulatory cells from two donors were kept in parallel and likewise analyzed with respect to methylation at day 0 as well as indicated time points. Shown are mean values±SEM. (B) FOXP3 expression in the CD25^hi^ and CD25^lo^ cells isolated at day 0 was determined by intracellular flow cytometry (left graph). During activation, FOXP3 expression was also determined in the sorted populations derived from stimulated CD25^lo^ cells (right graph). Data represent mean values from three separate donors±SEM. (C) CD25^hi^ and CD25^lo^ cells from three donors were isolated on day 5 post-activation. To examine the suppressive capacity of these activated CD25^lo^ derived cells, they were put into co-culture with autologous CFSE-stained non-activated CD4^+^ T cells in the presence of anti-CD3 and anti-CD28 antibodies, and autologous CD4^−^ cells as feeder cells. A control sample was included for each donor where only responder and feeder cells were included in the culture. Suppressive activity was evaluated on day 3 of co-culture as the mean fluorescence intensity ratio of CFSE^+^ responder cells in each sample relative the control sample.

In order to investigate whether the transient expression of FOXP3 during T cell activation was associated with *FOXP3* promoter demethylation, we analyzed male CD4^+^CD25^lo^ cells with respect to their methylation status during stimulation (Figure 6A). Cells were stimulated as previously described and DNA from the CD4^+^CD25^hi^ and CD4^+^CD25^lo^ fraction of the culture was extracted at day 5, 7, 12 and 16. The methylation status of the *FOXP3* promoter was analyzed using the restriction enzyme based method described above. In addition, stimulated CD4^+^CD25^hi^ cells isolated at day 0 was included in the experiment to show whether thymus derived T regulatory cells would maintain a demethylated FOXP3 promoter during the course of stimulation and expansion. Interestingly, an initial difference in the methylation level among the CD4^+^CD25^lo^ derived CD25^hi and lo^ cells was observed at day 5. The methylation level of the CD25^hi and lo^ CD4^+^CD25^lo^ derived cells gradually converged and levelled off at about 60% at day 16. Stimulated T regulatory cells however, maintained their demethylated profile although a slight increase in methylation could be seen. This is most probably explained by a small number of contaminating non-Tregs in the starting culture, which naturally respond more readily to stimuli and thus proliferate faster relative the Treg population.

FOXP3 expression was also measured using intracellular flow cytometry in all populations (Figure 6B). The highest levels of FOXP3 were observed in the stimulated Treg population. The CD25^hi^ fraction of the CD25^lo^ derived cells displayed higher levels of FOXP3 expression than the CD25^lo^ fraction throughout the course of stimulation.

The methylation level of the FOXP3 promoter region during transient expression was also determined in one donor using bisulphite sequencing (online supplemental material Figure S3A). Demethylation was detected after 2 and 12 days of culture (online supplemental material Table S1) in agreement with the results using the COBRA based technique (Figure 6A). On day 12 the bulk of cells we sorted into CD4^+^CD25^lo^ and CD4^+^CD25^hi^ populations and processed as previously described (online supplemental material Figure S3B). These populations displayed no significant difference in *FOXP3* promoter metylation.

### Suppressive ability of induced FOXP3^+^ cells

The transient expression of FOXP3 in activated T cells has been proposed as a mechanism of attenuating the activated state in these cells, but whether activated transient FOXP3 expressing T cells also have the capacity to suppress neighbouring cells is an unresolved matter [13]–[15], [17]. During the transient expression of FOXP3 we observe maximum MFI at day 5 of culture (Figure 5B). Therefore, this time point was chosen to evaluate the suppressive capacity of transient FOXP3 expressing cells. At day 5 of culture, the activated pool of cells contains a mixture of CD25^hi^ and CD25^lo^ cells with differing levels of methylation (Figure 6A) wherefore the suppressive capacity of these populations was measured separately. Suppressive capacity was determined based upon the ability to suppress the proliferation of CFSE stained CD4^+^ T cells. Higher suppressive capacity of the induced CD4^+^CD25^hi^ cells was observed compared to the CD4^+^CD25^lo^ sorted fraction (Figure 6C). This is also in agreement with the observation of higher FOXP3 expression among these cells (Figure 6B).

### Long-term follow-up after activation

**Figure 7:**
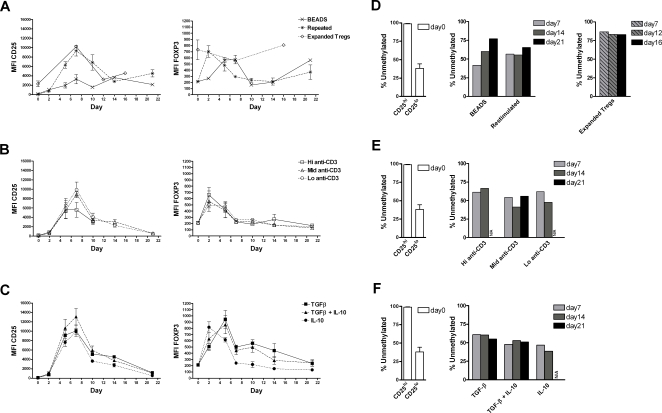
Activation results in a partial demethylation at long-term follow-up. CD25 and FOXP3 expression was monitored in CD4^+^CD25^lo^ cells during activation with (A) CD3/CD28 dynabeads as described in Figure 5 or repeated stimulation with anti-CD3 (5 µg/mL) and anti-CD28 (1 µg/mL) antibodies every 7 days. CD4^+^CD25^hi^ Tregs were also monitored as they were stimulated continuously with CD3/CD28 dynabeads. (B) CD4^+^CD25^lo^ cells stimulated with high (10 µg/mL), medium (5 µg/mL) or low (0.5 µg/mL) anti-CD3 antibody together with 1 µg/mL anti-CD28 antibody. (C) CD4^+^CD25^lo^ cells stimulated with anti-CD3 (5 µg/mL) and anti-CD28 (1 µg/mL) antibodies together with TGF-β (5 ng/mL), TGF-β (5 ng/mL) and IL-10 (10 ng/mL) or IL-10 only (10 ng/mL). Stimuli were removed on day 2 after stimulation and FACS analysis performed on days 0, 2, 5, 7, 10, 14 and 21. Data represent mean values from three separate donors±SEM, except for expanded Tregs where data represent mean values from two separate donors. Methylation status of the −77 reporter position was monitored during conditions described above (D–F). Evaluation of methylation status was performed on DNA from one donor at days 0, 7, 14 and 21 for each separate population. Methylation of expanded Tregs at day 7, 12 and 16 represent mean values from two separate donors.

CD4^+^CD25^hi^ cells isolated from peripheral blood are CD45RO^+^ and CD45Rb^lo^. They also have short telomere length consistent with memory T cells that have undergone several rounds of replication [31]. These observations together with the finding of antigen-specific Treg cells suggest that Tregs are generated as a result of time, after one or repeated antigen encounters. In order to investigate whether long-term culture after stimulus or repeated stimuli over time could affect the methylation pattern and/or FOXP3 expression in CD4^+^CD25^lo^ cells we followed the expression of CD25 and FOXP3 with FACS while monitoring the methylation status with the COBRA based method (figure 7A and D). Stimulation with CD3/CD28 Dynabeads or repeated stimulations with anti-CD3 and and-CD28 antibodies induced a transient expression of CD25 and FOXP3 and a gradual demethylation, similar to what was previously observed (Figure 6A and B). It is also worth noting that the Treg population remained FOXP3^hi^ throughout the stimulation.

It has been suggested that thymic derived T regulatory cells are generated as a result of an antigenic encounter that is insufficient to induce thymic deletion but sufficient to sustain a partial activation [32]. The generation of Tregs during suboptimal stimuli has also been suggested as a mechanism for inducing peripheral T regulatory cells. In order to investigate whether subimmunogenic conditions could be beneficial for a Treg like FOXP3 expression and methylation profile we stimulated CD4^+^CD25^lo^ cells with anti-CD3 antibody in three different concentrations together with anti-CD28 antibody. In all cases the stimulation resulted in a transient CD25 and FOXP3 expression and a partial decrease in methylation level as measured by the COBRA based method at the −77 reporter position (figure 7B & E). Cells were also stimulated with anti-CD3 or anti-CD28 alone. However, neither of these conditions induced any evident changes in FOXP3 expression nor promoter methylation (data not shown).

### Effect of TGF**-**β and IL-10 on methylation status

In mice, the conversion of CD4^+^CD25^−^ cells to the Treg phenotype is dependent on the presence of TGF-β [33]–[36]. Moreover it was very recently shown that TGF-β was able to induce demethylation at the mouse Foxp3 promoter during activation of CD4^+^CD25^−^ cells [27]. In order to investigate if TGF-β and/or IL-10, another Treg associated cytokine, have any effects on FOXP3 promoter methylation in the human context we stimulated CD4^+^CD25^lo^ cells with anti-CD3 and anti-CD28 antibodies in the presence of TGF-β and/or IL-10. We monitored CD25 and FOXP3 expression by flow cytometry and measured the methylation status of the FOXP3 promoter at given time points (day 0, 7, 14 and 21) using the COBRA based method previously described. Presence of TGF-β alone or together with IL-10 resulted in a more prolonged FOXP3 expression as compared to IL-10 only (figure 7C). However, no apparent difference in methylation status could be seen as compared to the activated population grown without the presence of TGF-β (figure 7F).

## Discussion

We have investigated the methylation status of a collection of tightly positioned conserved CpG dinucleotides in the *FOXP3* promoter region demonstrating a pattern which distinguishes the Treg population from induced FOXP3^hi^ T cells. Our data strongly suggests that the transient FOXP3 expression by activated T cells, observed by us and others, is a consequence of the methylation status at the *FOXP3* promoter region. Thus, only complete demethylation of the conserved FOXP3 promoter region support stable long term FOXP3 expression and a committed Treg phenotype in humans.

Mantel and colleagues have demonstrated that a conserved fragment of 348 bp upstream of the *FOXP3* transcription start site contains a minimal promoter necessary for induction of FOXP3 expression [11]. This region harbours important features of eukaryotic promoters such as TATA, GC and CAAT boxes. Disruption of these elements in reporter constructs resulted in reduced activity of the core promoter. Furthermore, the core promoter region contains functional binding sites for the transcription factors NFAT and AP-1, well known mediators of T cell activation [11]. We have analyzed the methylation status in a stretch of 8 tightly positioned CpG dinucleotides located between these transcription factor binding sites and the *FOXP3* transcription start site. Naturally occurring T regulatory cells displayed a completely demethylated promoter region while induced CD4^+^CD25^hi^ cells as well unstimulated and restimulated CD4^+^CD25^lo^ cells displayed a partially methylated promoter region. Interestingly, the partially methylated state was mostly pronounced at CpG position −77, which is fully conserved in cross species comparisons between human, mouse and rat. This emphasises the importance of the observed difference in methylation at this site.

Floess et al. [28] recently identified another differentially methylated region within the mouse *Foxp3* locus. A CpG rich region was found upstream of exon 1 displaying transcriptional activity and a differential methylation pattern between CD4^+^CD25^+^ and CD4^+^CD25^−^ cells. In a very recent report Kim et al [27] showed that this region binds cAMP response element binding protein (CREB) in a methylation dependent manner. In agreement with our data, the authors also showed a differential methylation state at the *Foxp3* promoter region between CD4^+^CD25^+^ and CD4^+^CD25^−^ cells and that treatment of CD4^+^CD25^−^ cells with 5-azacytidine results in elevated Foxp3 expression and demethylation at the *Foxp3* locus. Furthermore it was shown that addition of TGF-β to cell cultures induced demethylation at both the mouse promoter and the CREB binding region. Our experiments, on the other hand, show that addition of TGF-β during cell culture does not result in a Treg like demethylation at the human *FOXP3* promoter. In parallel to our study, another group have observed similar results regarding methylation in the conserved element upstream of exon 1 whitin the human *FOXP3* gene [37]. The observations of differing effects of TGF-β on methylation status in human or mouse settings agree with functional experiments conducted by others where it has been shown that TGF-β can induce functional Tregs in mice [33]–[36] but only transiently upregulates FOXP3 in humans and results in a non suppressive phenotype [38].

FOXP3 is used as a specific marker for the identification of T regulatory cells due to its correlation with suppressive function in freshly isolated CD4^+^CD25^+^ cells. However, as we and others have shown, FOXP3 is not restricted to naturally occurring Tregs but can be expressed by conventional T cells upon stimulation (Figure 4) [10]–[15], [17]. The suppressive capacity of the induced FOXP3 expressing cells has been tested with various results. In most cases, stimulation resulted in a transient FOXP3 expression and non suppressive function, however, suppressive phenotypes have been observed [10], [14]–[17], [39]. Regardless whether the variations in suppressive capacity of induced FOXP3 expressing cells are due to different stimulation conditions or due to donor dependent factors, it is now becoming increasingly accepted that stable FOXP3 expression is a prerequisite for a suppressive phenotype. We observed a significant difference in promoter methylation status between naturally occurring and induced CD4^+^CD25^hi^ cells. This implicates that the partially methylated state in the promoter region of CD4^+^CD25^lo^ and induced CD4^+^CD25^hi^ cells could be the cause for the transient FOXP3 expression in these cells. The restriction enzyme based screening method we describe provides a helpful tool for determination of Treg commitment as well as optimization of culturing conditions for Treg generation.

To illustrate how this method may be used, we measured FOXP3 expression in activated CD25^lo^ cells and correlated this to methylation level as well as suppressive capacity. A stimulated culture will after a certain time contain activated, not yet activated and formerly activated T cells, a fact which could explain the differing results obtained by others when analyzing these cells [13]–[15], [17]. To reduce the heterogeneity of the tested populations the activated T cells were sorted into CD25^hi^ and CD25^lo^ fractions throughout the course of stimulation prior to methylation analysis. A demethylated pattern was observed at an earlier stage in the sorted CD25^hi^ populations (Figure 6A), possibly due to FOXP3's linkage to expression of the IL-2R alpha chain (CD25) i.e. higher affinity to IL-2 and faster proliferative response. The association of proliferation to the gradual demethylation observed implies a cell division dependent passive demethylation process. It is interesting to note that the level of methylation in sorted CD25^hi and lo^, CD25^lo^ derived, populations converges and levels off at a partial demethylation around 60%. This convergence suggests that although the fast and slow responding cells within the CD25^lo^ population initially differed in methylation status, the induced CD25^hi^ cells retain their partially demethylated conformation as they downregulate CD25. In agreement with previous results, FOXP3 expression was higher in the CD25^hi^ population throughout the culture, and greater suppressive capacity was observed in these cells (Figure 6C).

In addition, we investigated the effect on methylation status of a spectrum of different stimuli and culturing conditions. Long-term follow-up after stimulation as well as subjection of CD4^+^CD25^lo^ cells to repeated stimuli revealed a partial demethylation of the −77 reporter position. The addition of TGF-β and/or IL-10 did not result in any evident additional demethylation. The restriction enzyme based method allowed us to speed up the analysis, and thus allowed inclusion of considerably more culture conditions and time points than would have been practically possible with conventional bisulphite sequencing.

The chromatin structure of the *FOXP3* promoter region in CD4^+^CD25^hi^, CD4^+^CD25^lo^ and naïve CD4^+^ cells has been shown to have an open configuration, as defined by histone H4 acetylation analysis [11]. This accessibility was further increased upon activation of CD4^+^CD25^−^ cells, an observation which suggested that CD4^+^CD25^−^ cells harbour a chromatin conformation which allows acquisition of a regulatory phenotype upon activation. Histone acetylation is a dynamic process, which provides a level of epigenetic regulation that is more likely to be affected during short term induction than CpG methylation [26]. Upon stimulation of CD4^+^CD25^lo^ cells demethylation occurs, although not reaching a fully demethylated pattern as we observe in FOXP3 expressing Tregs. These observations suggest that activated CD4^+^CD25^lo^ cells possess a chromatin structure which permits FOXP3 expression but is limited by the methylation status of the promoter. In developing immune cells, demethylation during cell fate decisions occurs either passively through exclusion of maintenance methylases from the replication fork [26], or actively as in the case of *IL-2* where a yet not identified enzyme is able to actively demethylate the promoter region upon TCR stimulation [40]. Our data shows that activation of CD4^+^CD25^lo^ cells results in partial demethylation of the human *FOXP3* promoter, and that the speed of demethylation seems to correlate to proliferation thus indicating passive demethylation. COBRA based analysis of the −77 reporter position opens up for screening of the methylation status in cell populations, and future experiments will reveal whether other stimuli than the ones investigated in this paper may result in full demethylation and stable FOXP3 expression.

The observations of variable suppressive capacity of Treg expansions [14] raise the question of which cells to use in expansion protocols. Possibly, only a hitherto unidentified subpopulation of circulating CD4^+^ cells posses a predisposed epigenetic architecture at the *FOXP3* locus which makes them suitable for expansions. Here, we identify the CpG position that best discriminates committed Tregs from transiently FOXP3 expressing CD4^+^ T cells, and introduce a method for convenient methylation screening of this site. In conclusion, we propose that the *FOXP3* promoter is under strict epigenetic control and that determination of the methylation status in the *FOXP3* promoter provides a more accurate definition of a suppressive phenotype in Tregs than FOXP3 expression alone. These considerations implicate CpG methylation analysis as a valuable tool for the distinction of committed human Tregs in both clinical and *in vitro* systems.

## Materials and Methods

### Isolation of Cell Populations

All procedures were done with approval from the Stockholm north regional ethic committee. Blood samples were drawn from healthy donors after written informed consent. CD4^+^ cells were purified using an autoMACS Separator (Miltenyi Biotec) and CD4^+^ T Cell Biotin-Antibody Cocktail and anti-biotin MicroBeads alternatively anti-human CD4 MicroBeads. For proliferation assay, this was followed by isolation of CD4^+^CD25^+^ cells with anti-CD25 MicroBeads. B cells were isolated with anti-CD19 PE antibodies (BD Biosciences PharMingen) and anti-PE microbeads (Miltenyi Biotec). CD4^+^CD25^hi^ and CD4^+^CD25^lo^ cells were isolated with the FACS Aria. All populations confirmed to be >95% pure. Cells in culture were kept at 37°C in RPMI medium (Invitrogen) containing 10% HuS, 1% PeSt, 1% Glutamine and 180 U rIL-2, alternatively AIM V medium (Invitrogen) +/− 10% FCS and 180 U rIL-2.

### Flow Cytometry

Peripheral blood mononuclear cells (PBMC) from healthy blood donors were purified from buffy coats using Ficoll-Paque Plus (Amersham Biosciences) according to manufacturer's protocol. Cells were labeled with surface antigen antibodies FITC-conjugated anti-CD4 and anti-CD19, and PE-conjugated anti-CD25 (BD Biosciences PharMingen), fixed and permeabilized using the FOXP3 staining buffer set (eBioscience) and stained with anti-FOXP3-APC according to manufacturer's protocol. Samples were run on either FACS Aria or FACS Calibur and analyzed with FACS Diva and Cell Quest Pro Software respectively.

### Stimulation of T cell cultures

CD4^+^CD25^lo^ cells isolated from male donors were activated for 48 hours with anti-CD3/CD28 Dynabeads (Invitrogen) (proportion cell∶beads 1∶1) in AIM-V containing 10% HUS, 180 U rIL-2 and 1% Penicillin Streptamycin (PeSt), after which the stimulus was removed and cultures followed for an additional 14 days of culture in AIM-V containing 10% HUS, 180 U rIL-2 and 1% PeSt. Alternatively, cells isolated as above were grown in RPMI containing 10% HUS, 180 U rIL-2 and 1% PeSt. In the specified experiments, cells were stimulated with anti-CD3 (5 µg/mL) and anti-CD28 (1 µg/mL) antibodies in the presence of 5 ng/mL TGF-β and/or 10 ng/mL IL-10. For evaluation of the effect of suboptimal versus conventional and high TCR-stimulus strength 10 µg/mL, 5 µg/mL and 0.5 µg/mL anti-CD3 antibody was used in combination with 1 µg/mL anti-CD28, as well as separate cultures with anti-CD3 (5 µg/mL) or anti-CD28 (1 µg/mL) only.

### T Cell Proliferation Assay

Suppressive capacity was investigated either by incorporation of [^3^H]Thymidine or CFSE staining. For thymidine incorporation, 5*10^4^ CD4^−^ cells irradiated with 25 Gy were combined with 3*10^4^ CD4^+^CD25^−^ responder cells and 1 µg/mL soluble CD28 in 96-well plates coated with 4 µg/mL anti-CD3 IgG. CD4^+^CD25^+^ cells were added to the wells in relations varying from 1∶1 to 1∶4 to the CD4^+^CD25^−^. Equal numbers of CD4^+^CD25^−^ cells were added to the responder cells in an additional set of wells as control. All samples run in triplicate. Plates kept at 37°C for four or five days, pulsed with [^3^H]Thymidine for 18 h and frozen at −20°C. Cells were thawed and the well content transferred to a glass fibre filter (Wallac) by a cell harvester (TOMTEC). MeltiLex A – Melt-on scintillator sheets (Wallac) were used for detection of radioactivity which was measured using a 1205 Betaplate Liquid Scintillation Counter (Wallac).

The composition of the CFSE assays was as above except that no cells were irradiated and the responder population was stained with CFSE prior to setup. Proliferation was evaluated on day 4 as a decrease of MFI within the positive population in FL-1 with responder cells only as positive control.

### RNA isolation and Reverse Transcriptase Quantitative Real time PCR

RNA extraction was performed with the RNA Mini Kit (Bio-Rad) alternatively with the use of TRIZOL reagent (Invitrogen), and cDNA synthesis was carried out with iScript cDNA Synthesis Kit (Bio-Rad) according to the manufacturer's protocol. Quantitative PCR was performed on an iCyclerIQ, and cycle thresholds were obtained using iCycler IQ™ Optical System Software Version 3.1 from BIO-RAD. There have been several reports about the questionable reliability of different housekeeping genes [41], [42]. In an extensive study by Radonic et al [42] RNA polymerase II (RPII) was found to be the most reliable in a wide variety of cell types including lymphocytes and thus this was our choice of housekeeping gene. Expression levels were normalized to RPII using the 2^−ΔΔCt^ method. Primers were obtained from Cybergene AB (Table 2). For ordinary PCR, ThermoPol Reaction Buffer and Taq DNA polymerase (New England Biolabs) was used whereas iQSYBR Green Supermix (Bio-Rad) was used in quantitative real time PCR. The reactions were carried out in MyCycler Thermal Cycler or iCycler iQ Real-Time PCR Detection System (Bio-Rad).

**Table 2:** Primer sequences.

*Name*	*(Sequence 5′⇒ 3′)*	*Purpose*
FOXP3 forward	CAG CAC ATT CCC AGA GTT CCT C	QT PCR
FOXP3 reverse	GCG TGT GAA CCA GTG GTA GAT C	QT PCR
RPII forward	GCA CCA CGT CCA ATG ACA T	QT PCR
RPII reverse	GTG CGG CTG CTT CCA TAA	QT PCR
FOXP3 bilsulphite forward	TGG TGA AGT GGA TTG ATA GAA AAG G	Bisulphite sequencing
FOXP3 bisulphite reverse	TAT AAA AAC CCC CCC CCA CC	Bisulphite sequencing
FOXP3 COBRA Forward	TTG GAT TAT TAG AAG AGA GAG GTT	COBRA
FOXP3 COBRA Reverse	6-FAM CTA ACA AAA AAA AAT CAA CCT AAC	COBRA

### DNA isolation and methylation analysis

Methylation analysis was carried out using bisulphite sequencing. Genomic DNA from cell populations isolated from male donors was extracted using DNeasy Blood & Tisse Kit (QIAGEN) and bisulphite converted using EZ DNA Methylation Kit (ZYMO Research) according to the manufacturer's instruction. This reaction converts all non-methylated cytosine bases into uracil. The FOXP3 promoter region was amplified by PCR using primers shown in Table 2 and PCR products were cloned into a pCR®4-TOPO® vector using a TOPO TA Cloning® Kit (Invitrogen). DNA from individual bacterial clones was then included in sequencing reactions with T3 and T7 primers using BigDye® Terminator v1.1 Cycle Sequencing Kit (Applied Biosystems) according to manufacturer's instruction. Samples were examined on a 310 Genetic Analyzer (Applied Biosystems) and resulting sequences analyzed using ABI Prism Sequencing Analysis Software 3.7 (Applied Biosystems).

### Bioinformatics

Information regarding conserved regions in the FOXP3 promoter was acquired from the UCSC Genome Bioinformatics Site (http://genome.ucsc.edu/), and analyzed with regard to CpG dinucleotides using the program ApE v1.10.1. Sequence alignment for conservation analysis was performed using m-Vista genome browser (http://genome.lbl.gov/vista/index.shtml) and ClustalW (http://www.ebi.ac.uk/clustalw/). Statistical analysis of obtained data was performed using Fisher's exact test.

### Combined Bisulphite Restriction Enzyme Analysis (COBRA)

Genomic DNA was isolated and bisulphite treated as previously described. The FOXP3 promoter region was amplified by PCR with a nonconjugated forward primer and a 5′ 6-FAM conjugated reverse primer shown in Table 2. PCR products were digested with Aci1 in excess. Following a phenol chlorofom extraction digested products were loaded onto a 310 Genetic Analyzer (Applied Biosystems) and fragment analysis was performed using Gene Scan v3.7 application software (Applied Biosystems). As controls for validity of the COBRA reaction M.Sss1 (New England Biolobs) treated DNA from a bacterial artificial chromosome (BAC) containing the FOXP3 locus (RP11-528A24, BACPAC Resources, CHORI) and unmodified BAC DNA were used.

## Supporting Information

Table S1 P-value for each CpG position during T cell activation.
(0.03 MB DOC)

Figure S1 Conservation of the *FOXP3* promoter region. ClustalW alignment indicating cross species conservation in the *FOXP3* promoter region. Stars under each nucleotide position indicate full conservation. Aligned score indicate cross species conservation in percent.
(0.96 MB TIF)

Figure S2 Conservation of the *FOXP3* promoter CpG containing region. ClustalW alignment as described in Figure S1 including only the CpG containing region of the *FOXP3* promoter.
(0.51 MB TIF)

Figure S3 Methylation of the *FOXP3* promoter during stimulation of CD4^+^CD25^lo^ cells from one male donor. (A) CD4^+^CD25^lo^ cells were stimulated with CD3/CD28 dynabeads as described in Figure 5 and DNA extracted at day 0, 2 and 12 for bisulphite sequencing. Figures illustrating methylation at indicated sites as previously described in Figure 3. (B) Methylation status of CpG positions of sorted CD4^+^CD25^lo^ and CD4^+^CD25^hi^ cells on day 13 of culture.
(1.15 MB TIF)
