# Supplementary material for: FOXP3 Promoter Demethylation Reveals the Committed Treg Population in Humans
Source: PLoS One. 2008 Feb 20;3(2):e1612. doi: 10.1371/journal.pone.0001612 (PMC2238816; doi:10.1371/journal.pone.0001612)
Supplement: Table S1 — P-value for each CpG position during T cell activation. (0.03 MB DOC) [file pone.0001612.s001.doc]

**Table S1.** P-value for each CpG position during T cell activation.

|  | *-138* | *-126* | *-113* | *-77* | *-65* | *-58* | *-43* | *-15* |
| --- | --- | --- | --- | --- | --- | --- | --- | --- |
| *d0 vs d2* | 0.20 | 0.10 | <0.05 | 0.27 | <0.05 | 0.064 | 0.56 | 0.21 |
| *d0 vs d12* | 0.09 | <0.01 | <0.001 | 0.20 | <0.05 | <0.05 | 0.055 | 0.054 |
