# Supplementary figures and images for: FOXP3 Promoter Demethylation Reveals the Committed Treg Population in Humans
Source: PLoS One. 2008 Feb 20;3(2):e1612. doi: 10.1371/journal.pone.0001612 (PMC2238816; doi:10.1371/journal.pone.0001612)

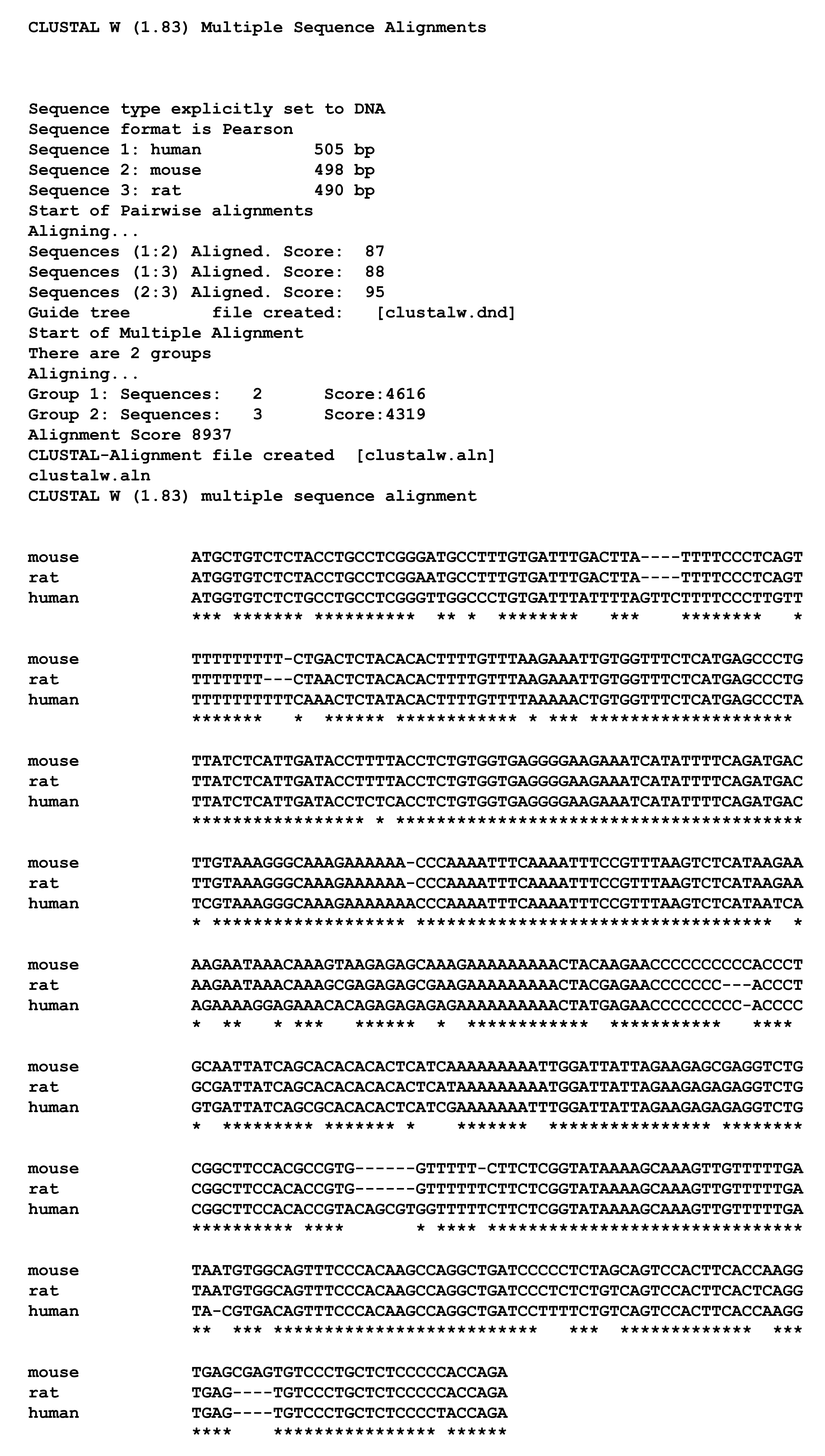

Supplement: Figure S1 — Conservation of the FOXP3 promoter region. ClustalW alignment indicating cross species conservation in the FOXP3 promoter region. Stars under each nucleotide position indicate full conservation. Aligned score indicate cross species conservation in percent. (0.96 MB TIF) [file pone.0001612.s002.tif]

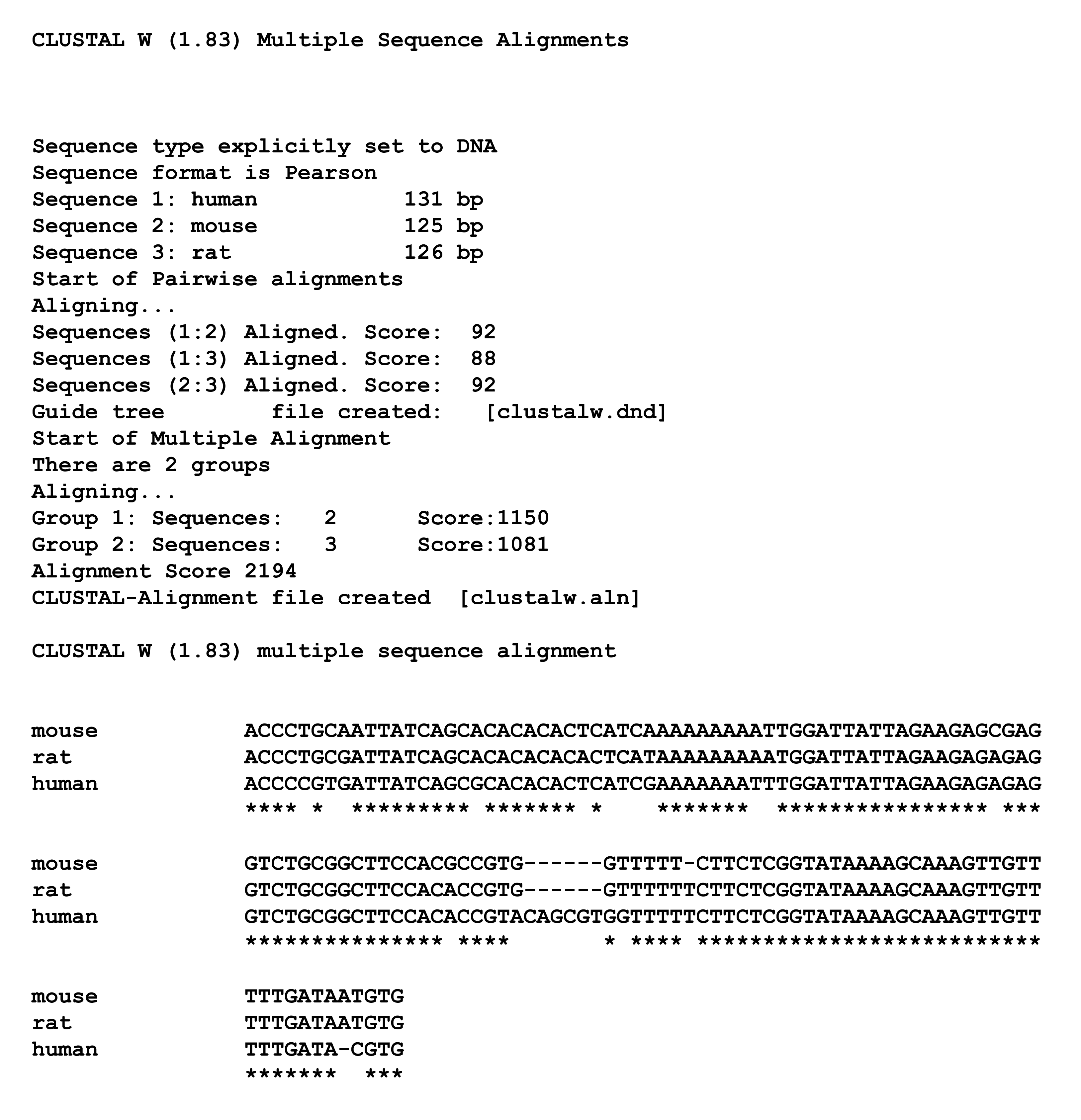

Supplement: Figure S2 — Conservation of the FOXP3 promoter CpG containing region. ClustalW alignment as described in Figure S1 including only the CpG containing region of the FOXP3 promoter. (0.51 MB TIF) [file pone.0001612.s003.tif]
